# Curcumin and Emodin Down-Regulate TGF-β Signaling Pathway in Human Cervical Cancer Cells

**DOI:** 10.1371/journal.pone.0120045

**Published:** 2015-03-18

**Authors:** Pooja Chandrakant Thacker, Devarajan Karunagaran

**Affiliations:** Department of Biotechnology, Bhupat and Jyoti Mehta School of Biosciences, Indian Institute of Technology Madras, Chennai 600036, India; University of Quebec at Trois-Rivieres, CANADA

## Abstract

Cervical cancer is the major cause of cancer related deaths in women, especially in developing countries and Human Papilloma Virus infection in conjunction with multiple deregulated signaling pathways leads to cervical carcinogenesis. TGF-β signaling in later stages of cancer is known to induce epithelial to mesenchymal transition promoting tumor growth. Phytochemicals, curcumin and emodin, are effective as chemopreventive and chemotherapeutic compounds against several cancers including cervical cancer. The main objective of this work was to study the effect of curcumin and emodin on TGF-β signaling pathway and its functional relevance to growth, migration and invasion in two cervical cancer cell lines, SiHa and HeLa. Since TGF-β and Wnt/β-catenin signaling pathways are known to cross talk having common downstream targets, we analyzed the effect of TGF-β on β-catenin (an important player in Wnt/β-catenin signaling) and also studied whether curcumin and emodin modulate them. We observed that curcumin and emodin effectively down regulate TGF-β signaling pathway by decreasing the expression of TGF-β Receptor II, P-Smad3 and Smad4, and also counterbalance the tumorigenic effects of TGF-β by inhibiting the TGF-β-induced migration and invasion. Expression of downstream effectors of TGF-β signaling pathway, cyclinD1, p21 and Pin1, was inhibited along with the down regulation of key mesenchymal markers (Snail and Slug) upon curcumin and emodin treatment. Curcumin and emodin were also found to synergistically inhibit cell population and migration in SiHa and HeLa cells. Moreover, we found that TGF-β activates Wnt/β-catenin signaling pathway in HeLa cells, and curcumin and emodin down regulate the pathway by inhibiting β-catenin. Taken together our data provide a mechanistic basis for the use of curcumin and emodin in the treatment of cervical cancer.

## Introduction

Cervical cancer is the fourth leading cause of cancer related deaths in women worldwide and more than 85% of cervical cancer cases and deaths occur in developing countries out of which, India is reported to account for 27% of the total cervical cancer deaths [1]. The underlying mechanism promoting cervical tumorigenesis is complex and includes deregulation of key signaling pathways apart from the major role played by HPV (Human Papilloma Virus) infection [2]. TGF-β signaling pathway is implicated in complex cellular processes regulating development, differentiation and homeostasis [3]. TGF-β ligand binds to TGF-β receptor II, activating TGF-β receptor I by transphosphorylation, that in turn activates R-Smads (Smad2 and Smad3) via phosphorylation at their C-terminal residues. Activated R-Smads form a heterocomplex with Smad4 and translocate to the nucleus where they activate TGF-β responsive genes [4]. In the early stages of tumorigenesis, TGF-β signaling pathway acts as a tumor suppressor preventing progression of cell cycle through G_1_ phase by the down regulation of CyclinD1 and Cyclin dependent kinase (CDK) proteins and induction of p15^INK4B^, p16^INK4A^, which inhibit CDK4 and CDK6; likewise p21^Cip1^or p27^Kip1^appears to fulfill the function of p15^INK4B^ in its absence [5, 6]. TGF-β-mediated apoptosis is known to increase the ratio of expression of proapoptotic Bax and anti-apoptotic Bcl-2 proteins [7]. However, in advanced stages of cancer, TGF-β signaling is also shown to promote invasiveness and metastasis by inducing the expression of Snail and other transcription factors thereby causing differentiation of epithelial to mesenchymal phenotype [8]. Slug and N-cadherin, known players of EMT, induced by TGF-β are involved in migration and invasion [9], and TGF-β-mediated induction of N-cadherin involves Pin1 (peptidyl-prolyl cis/trans isomerase), known to play an important role in TGF-β-induced migration and invasion of cancer cells [10]. TGF-β is also shown to stimulate cyclinD1 expression at least in part through activation of Wnt/β-catenin signaling [11]. Wnt/β-catenin signaling is known to regulate broad range of cellular processes that regulate the ability of the multifunctional β-catenin protein to activate the transcription of genes involved in cell adhesion, proliferation, differentiation, and other signaling pathways [12]. Deregulation of Wnt/β-catenin signaling is known to influence carcinogenesis, and alterations in Wnt/β-catenin signaling pathway are reported in cervical neoplasia [13]. Wnt ligand binds to the transmembrane frizzled receptors, stabilizing β-catenin by inhibiting the activity of glycogen synthase kinase 3 β (GSK-3 β), associated with a multimeric death complex consisting of axin, adenomatosis polyposis coli (APC) and casein kinase 1α (CK1α), wherein CK1α and GSK-3β phosphorylate β-catenin sequentially, marking it for ubiquitination and proteasomal degradation. In response to activated Wnt/β-catenin signaling, GSK-3 β is inhibited by disheveled proteins, whereby, β-catenin accumulates in the cytoplasm and translocates into the nucleus. In the nucleus, β-catenin in association with T-cell factor/lymphocyte enhancer factor (Tcf/Lef) family and other transcriptional cofactors, activates a variety of target genes including c-myc and cyclin D1 [14]. Convergence of TGF-β and Wnt/β-catenin signaling promotes epithelial to mesenchymal transition by inducing the expression of Snail and Slug [15]. There is a marked increase in the expression of TGF-β mRNA and protein in human cancers and high expression of TGF-β correlates with more advanced stages of malignancy and decreased survival [16]. Cervical cancer cells are known to secrete TGF-β [17], which is capable of augmenting intratumoral stroma and decreasing tumor infiltrate, enhancing tumor growth and metastasis while evading host’s immune system [18]. The pivotal role of TGF-β in promoting tumor progression suggests that the signaling pathway may be a good target for therapy of cancer [16]. The current therapeutic options for cervical cancer such as radiotherapy, surgery and chemotherapy, especially in the developing countries, have their limitations mainly due to the unavailability of the treatment modality, the cost, severity of side effects, high systemic toxicity and drug resistance, and frequent development of infertility post therapy [19]. Moreover, increasing resistance to chemotherapy emerging out of deregulation of signaling cascades is a major challenge towards effective treatment, and TGF-β signaling is one of the signaling pathways known to cause chemo-resistance via induction of EMT [20]. Chemopreventive phytochemicals target various intracellular signaling cascades to inhibit tumor promotion, proliferation and progression [21]. Curcumin, a polyphenol derived from *Curcuma longa* and emodin, an anthraquinone present in roots and barks of several medicinal plants are thought to act by modulating the deregulated signaling pathways in neoplastic cells [22, 23]. Curcumin is known to induce apoptosis and block the progression of cervical cancer [24–26] and emodin is also shown to induce apoptosis in cervical cancer cells [27, 28]. Curcumin is known to inhibit TGF-β signaling in breast and pancreatic cancers [29, 30] while emodin is known to differentially regulate TGF-β signaling in a context dependent manner [31, 32], however, the effect of these phytochemicals on TGF-β signaling in cervical cancer cells remains to be studied.

Thus, the main objective of this work was to study the effect of curcumin and emodin on TGF-β signaling pathway and its functional relevance to growth and migration in two cervical cancer cell lines, SiHa and HeLa. Further, it was of interest to study if the combined effect of these compounds had any synergistic or additive effects. We observed that curcumin and emodin down regulate TGF-β signaling pathway in SiHa and HeLa cells, and also counterbalance the tumorigenic effects of TGF-β by inhibiting TGF-β-induced migration and invasion of these cells. Also, the combination of curcumin and emodin synergistically inhibited cell viability in SiHa and HeLa cells.

## Materials and Methods

### Reagents

Curcumin, emodin, 5, 5´, 6, 6´-tetrachloro-1, 1´, 3, 3´-tetraethylbenzimidazol-carbocyanine iodide (JC-1) were purchased from Sigma (St. Louis, MO; USA). A 10 mM solution of each compound (curcumin or emodin) was prepared in dimethyl sulfoxide (DMSO), stored as small aliquots at–20°C, and then diluted further in cell culture medium as needed. DMSO (0.4%) was used as vehicle control for all the experiments. Human TGF-β1was purchased from Peprotech (USA). Geltrex, Propidium iodide, Lipofectamine 2000, Dulbecco’s Modified Eagle Medium (DMEM), Fetal bovine serum (FBS South American origin) were purchased from GIBCO (Invitrogen, Carlsbad, CA; USA). Polyethylenimine (PEI) was purchased from Polysciences (Warrington, PA, USA). Polyvinylidene fluoride membrane and Enhanced chemiluminescence kit were obtained from BIORAD (Hercules, CA; USA). Protease and phosphatase inhibitor cocktail was purchased from Sigma (St. Louis, MO; USA). β-Actin antibody was purchased from Sigma-Aldrich (St. Louis, MO; USA) and antibodies to TGF-β receptor I, TGF-β receptor II, Smad4, N-cadherin, Pin1, β-catenin, GSK3β and P-GSK3β (ser9) were obtained from Santa Cruz (CA; USA). Antibodies to P-Smad2, Smad2, P-Smad3, Smad3, Snail, CyclinD1, Slug, Bax, Bcl-2 and p15, p16, p21, p27 were from Cell Signaling (Danvers, MA; USA) and horseradish peroxidase conjugated secondary antibody was obtained from Jackson ImmunoResearch Inc. (USA).

### Cell culture

Human cervical cancer cell lines, HeLa and SiHa, were obtained from the National Centre for Cell Sciences (NCCS) Pune, India. They were cultured in complete Dulbecco’s Modified Eagle Medium (cDMEM) composed of DMEM supplemented with streptomycin (100 μg/ml), penicillin (100 U/ml) and 10% FBS. Cells were maintained in a humidified incubator at 37^0^C with 5% CO_2_.

### Resazurin reduction assay

Analysis of cytotoxicity was determined by resazurin reduction assay [33]. Cells were seeded in a 96well plate at a density of 5000 per well and grown overnight. Cells were then treated with different concentrations of curcumin and emodin with or without 5ng/ml of TGF-β in cDMEM for 48h. Resazurin dye was added on to the media at a final concentration of 0.1mg/ml, incubated for 3h for the reduction of the blue dye resazurin to pink resorufin and the absorbance was measured at 570 and 595nm. The data were analyzed as percent of control, where the control wells were treated with equivalent amounts of DMSO alone. The analyzed data representing percentage population of cells, were plotted as average ± standard error of mean (S.E.M.). IC_50_ was obtained by determining the concentration of compounds resulting in 50% inhibition of cell population after 48h of treatment by using GraphPad PRISM software (GraphPad Software, Inc.).

### JC-1 staining

JC-1 (5,5′, 6,6′-tetrachloro-1,1′,3,3′-tetraethylbenzimidazolcarbocyanine iodide) is a lipophilic fluorescent cationic dye used to assess the mitochondrial membrane potential (ΔΨm) in cells [34]. Intact ΔΨm exhibits negative charge, allowing the JC-1 dye to enter the mitochondrial matrix where it accumulates forming aggregates that fluoresce red while the mitochondrial membrane collapses in the apoptotic cells, and thus JC-1 cannot accumulate in mitochondria of these cells, wherein it remains in cytoplasm in the monomeric form that fluoresce green. The decrease in the ratio of red to green fluorescence of the dye is an indication of the decreased ΔΨm of cells. After treatment of curcumin and emodin in presence or absence of TGF-β for 48h, the JC-1 containing medium (final JC-1 concentration −5 μg/mL) was added to the cells, followed by incubation for 20 min at 37°C. Subsequently the cells were washed once with PBS and the fluorescence was measured at excitation: 485; emission: 535 for monomer form (green fluorescence) and excitation 550; emission 600 for the aggregates (red fluorescence), using Perkin Elmer Enspire multi-plate reader. Ratio of red to green fluorescence was analyzed and plotted against appropriate treatment conditions.

### Wound healing assay

Changes in migration of cells upon treatment were studied using wound healing assay. Cells were grown as a monolayer in a 48 well plate and at 100% confluence; a scratch was made on the monolayer. The cells were gently washed and treated with curcumin and emodin in presence or absence of TGF-β and maintained in serum free medium. Cell migration into the wound surface was monitored by microscopy. Images of the scratch were taken at 4X magnification immediately after addition of emodin and after 48h. The extent of migration in each sample was measured as the area covered by the cells in 48h using Tscratch analysis software [35] and expressed as percentage of control.

### Matrigel Invasion assay

Cervical cancer cell invasion was assessed by Boyden assay using transwell chambers (8 μm pore size; Costar, MA, USA). Chambers were coated with 100 μl matrigel and incubated overnight. Serum-starved SiHa (25,000) and HeLa (50,000) cells were seeded in the upper chambers of the Matrigel-coated wells (BD Biosciences, San Diego, CA, USA) containing 100 μl DMEM plus the desired treatment. Curcumin or emodin containing DMEM with or without TGF-β, supplemented with 10% FBS was placed in the lower chambers, and cells were allowed to migrate through the filter for 48 h. Cells that failed to migrate were removed from the upper surface of the chambers by scraping with a cotton swab. Cells that successfully migrated through the matrigel and the membrane barrier to the lower membrane were fixed in 100% methanol and stained with 2% crystal violet. Images of the stained cells were captured at 10X magnification. The extent of invasion was expressed as percentage of control deduced from the absorbance of crystal violet released by lysing the cells with 10% acetic acid at 595nm.

### Cell cycle analysis

Propidium iodide staining and flow cytometry were used to assess the cell cycle distribution profile. The treated cells were washed with PBS EDTA and then harvested using 0.25% Trypsin EDTA and suspended in cDMEM. Cells were then washed with PBS and centrifuged at 1200 rpm at 4°C for 5min, and resuspended in 300 μL PBS, fixed with 0.7ml 70% ethanol overnight. Fixed cells were spun down, washed with 0.1% FBS containing PBS and then suspended in 300 μL propidium iodide (2μg/mL) staining solution with RNase A (200 μg/mL) in the dark, incubated at 37°C for 1h. Data from 10,000 cells were collected for each sample. Data acquisition and analysis were performed on a flow cytometer (Beckman Coulter cell lab Quanta).

### SBE and TOPFlash luciferase reporter assay

Cells (30,000/well) were seeded in 96 well plates and co-transfected with SBE4-Luc reporter construct, containing four copies of the Smad binding site (GTCTAGAC) cloned into pBV-Luc indicative of the downstream transcriptional activation of TGF-β signaling pathway (Addgene plasmid 16495) [36] or TOPFlash, a luciferase reporter of β-catenin-mediated transcriptional activation, with 7 TCF/LEF binding sites upstream of luciferase (Addgene plasmid 12456) [37]and pRL-TK (renilla luciferase) plasmid using PEI (for SBE Luciferase construct) and lipofectamine 2000 (for TOPFlash luciferase construct) according to the manufacturer’s instructions, in serum and antibiotic free DMEM. Six hours after transfection, the medium was changed and cells were treated with curcumin and emodin in presence or absence of TGF-β in cDMEM. After 24h, the cells were lysed and firefly and renilla luciferase activities were assayed [38].

### Protein isolation and western blotting

Cells were washed with phosphate-buffered saline (PBS) 48h post treatment, and total protein was extracted after scraping and collecting cells in radio immuno precipitation assay (RIPA) lysis buffer [20mM Tris (pH 7.5), 150mM sodium chloride, 1mM ethylene diamine tetra acetic acid, 1mM β-glycerophosphate, 1% Triton X 100, 2.5mM Sodium pyrophosphate, 1mM sodium orthovanadate, 0.5% sodium deoxycholate, 1mM phenyl methane sulfonyl fluoride, 20mM sodium fluoride, 1% protease inhibitor], incubated for 1h, and centrifuged. Total cell protein in the supernatant was estimated using Bradford assay, and 30μg of protein was subjected to SDS-PAGE separation followed by transfer onto polyvinylidenedifluoride membrane. The membrane was incubated with primary antibodies against TGF-β receptor I, TGF-β receptor II, P-Smad2, Smad2, P-Smad3, Smad3, Smad4, β-catenin, GSK3β, P-GSK3β (ser9), N-cadherin, Pin1, Snail, CyclinD1, Slug, Bax, Bcl-2, p15, p16, p21, p27, CDK6 (1:1000 dilution), or β-Actin (1:10,000 dilution) overnight, followed by incubation with an HRP-conjugated secondary antibody. Protein bands were detected using an enhanced chemiluminescence detection kit and visualized by using the Versa Doc image analysis system (Bio-Rad).

### Statistical analysis

Two tailed unpaired student’s T-test or two-way analysis of variance was used for statistical analysis of the untreated and treated samples or as indicated. P values < 0.05, 0.01and 0.001 are indicated by ‘*’ ‘**’ and ‘***’, respectively.

## Results

### Curcumin and emodin induce cytotoxicity in the presence or absence of TGF-β in human cervical cancer cells

**Fig 1:**
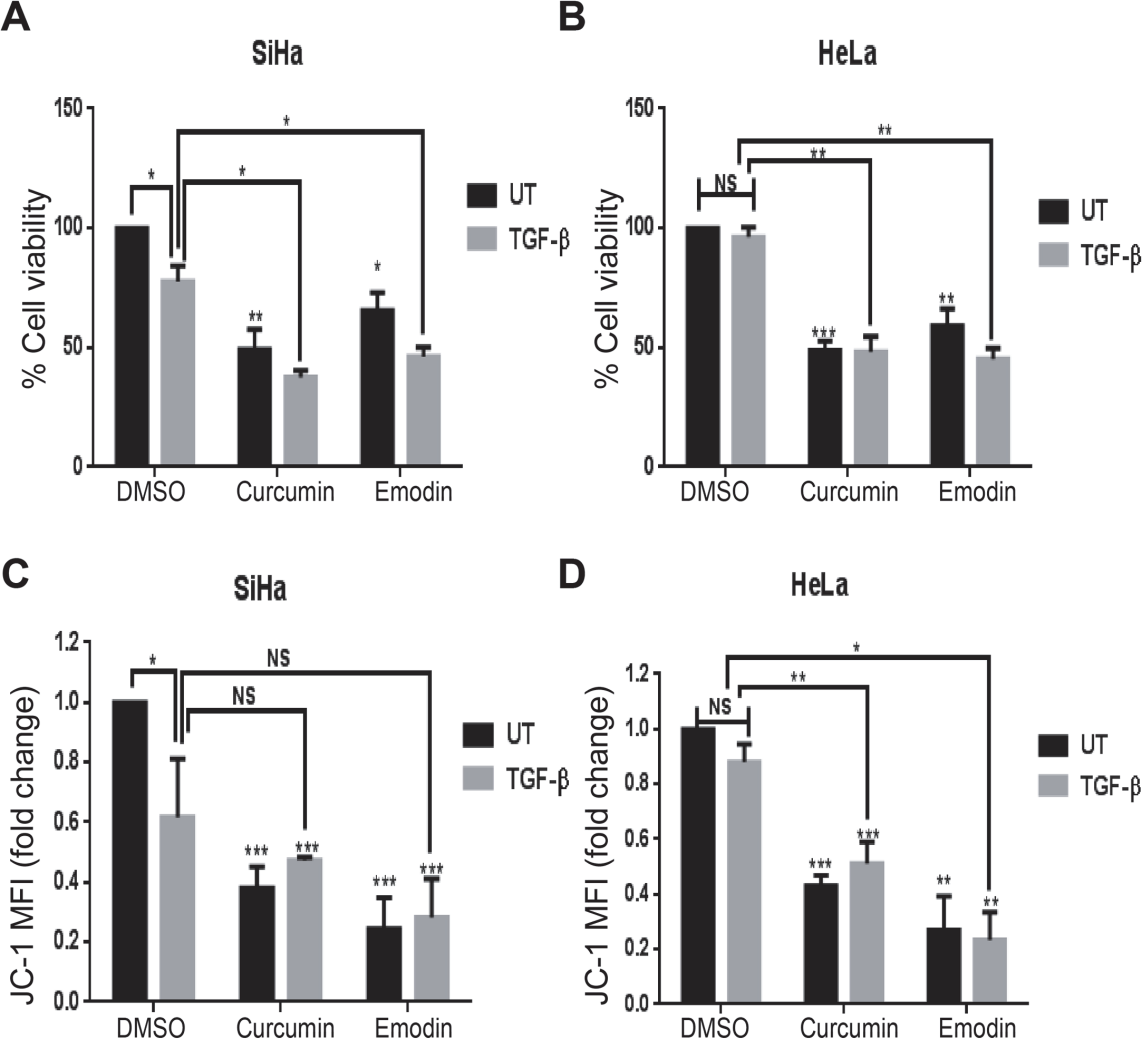
Effects of TGF-β, curcumin and emodin on cell viability and mitochondrial membrane potential. SiHa (A) and HeLa (B) cells were treated with 15 and 25μM curcumin, respectively or 40 μM emodin in the presence (TGF-β) or absence (UT) of 5ng/ml TGF-β for 48h and assayed for cell viability by resazurin reduction method. Percentage cell viability was calculated by normalizing the absorbance of treated samples against the DMSO control (n = 3, mean ± S.E.M.). Curcumin or emodin treated SiHa (C) and HeLa (D) cells in presence (TGF-β) and absence (UT) of TGF-β were assayed for JC-1 activity using a fluorimetric method as described in Materials and Methods. Results were plotted as fold change of mean fluorescent intensity (MFI) with respect to DMSO control (n = 3, mean ± S.E.M.).

We have tested the effects of various concentrations of curcumin and emodin on the viabilities of human cervical cancer cells and the IC_50_ values calculated from these data are listed in Table 1. Curcumin was relatively more cytotoxic to human cervical cancer cells analyzed in this study (Table 1) and it was used at 15μM in SiHa and 25μM in HeLa cells, while emodin was used at 40μM in SiHa and HeLa cells for further experiments. Since secretion of TGF-β into the stroma is often observed during the advanced stages of cervical cancer [18], we wanted to study the effect of these compounds in the presence of TGF-β. In line with our earlier report, 5ng/ml TGF-β decreased the cell population [39] and inhibited mitochondrial membrane potential in SiHa cells (Fig. 1A and C), while HeLa cells remained unaffected (Fig. 1B and D). Curcumin and emodin were found to significantly inhibit cell growth (Fig. 1A and B) as observed by resazurin reduction assay in the presence as well as absence of TGF-β in these cells. The decrease in the mitochondrial membrane potential (Fig. 1C and D) brought about by curcumin or emodin as analyzed by JC-1 dye fluorimetry was significant in SiHa and HeLa cells. However, this effect was independent of TGF-β in HeLa but was not significant in presence of TGF-β in SiHa cells (Fig. 1C and D). These results indicate that although TGF-β decreased the cell growth and mitochondrial membrane potential in SiHa but not in HeLa cells, it did not affect curcumin or emodin-mediated inhibition of growth and mitochondrial membrane potential in these cells.

**Table 1:** IC_50_ of curcumin and emodin in human cervical cancer cells.

Cell lines	IC_50_ for curcumin (μM)	IC_50_ for emodin (μM)
SiHa	15 ± 6.8	45 ± 3.4
HeLa	25 ± 5.2	40 ± 4.9

SiHa and HeLa cells treated with different concentrations of curcumin and emodin were assayed for cell viability by resazurin reduction method at 48h and IC_50_ values were calculated as described in Materials and Methods. (n = 3, mean ± S.E.M.)

**Fig 2:**
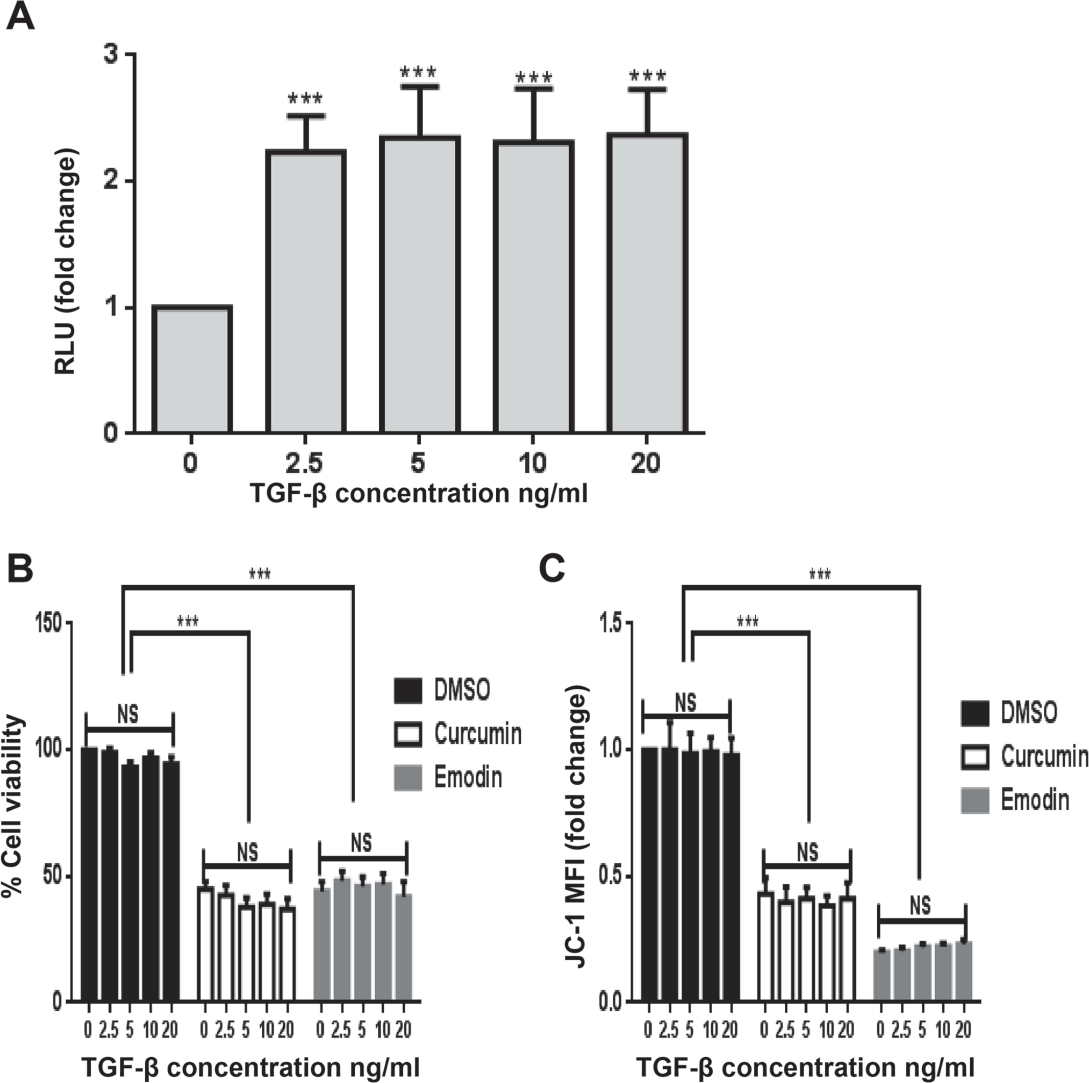
Effect of different concentrations of TGF-β on cell viability and mitochondrial membrane potential. HeLa cells (A) transfected with SBE firefly luciferase and TK-renilla luciferase constructs were treated (6h post transfection) with 2.5, 5, 10 and 20ng/ml of TGF-β and assayed for luciferase activity 24h post treatment. Transfection efficiency was normalized by calculating the relative ratio of firefly to renilla luciferase activities and the results were plotted as fold change with respect to the untreated control. HeLa cells were treated with different concentrations of TGF-β in presence and absence of curcumin or emodin and assayed for cell viability by resazurin reduction method (B), and mitochondrial membrane potential by analyzing JC-1 activity (C) using a fluorimetric method after 48h as described in Materials and Methods (n = 3, mean ± S.E.M.).

Since 5ng/ml TGF-β did not have any significant effect on the cell viability or mitochondrial membrane potential in HeLa cells, whether a different concentration of TGF-β would have similar or different response was examined. In this regard, HeLa cells were treated with different concentrations of TGF-β and its effect on its signaling was assessed by analyzing the activity of TGF-β-responsive SBE-Luc reporter construct, in a read-out assay. It was observed that 2.5 ng/ml of TGF-β significantly induced the SBE luciferase activity but there was no significant difference in the extent of induction with increase in its concentration (Fig. 2A). Further, HeLa cells treated with different concentrations of TGF-β in presence or absence of curcumin or emodin were assessed for changes in cell viability and mitochondrial membrane potential. Different concentrations of TGF-β had no significant effect on cell viability (Fig. 2B) or mitochondrial membrane potential (Fig. 2C), and it did not influence the inhibition of cell viability (Fig. 2B) or mitochondrial membrane potential (Fig. 2C) by curcumin and emodin in these cells. These results indicate that lack of responsiveness of HeLa cells to TGF-β towards cell viability and mitochondrial membrane potential was concentration independent, and also TGF-β did not affect the growth inhibitory activity of curcumin or emodin.

### Curcumin and emodin inhibit cell growth, migration and invasion in the presence or absence of TGF-β in SiHa and HeLa cells

**Fig 3:**
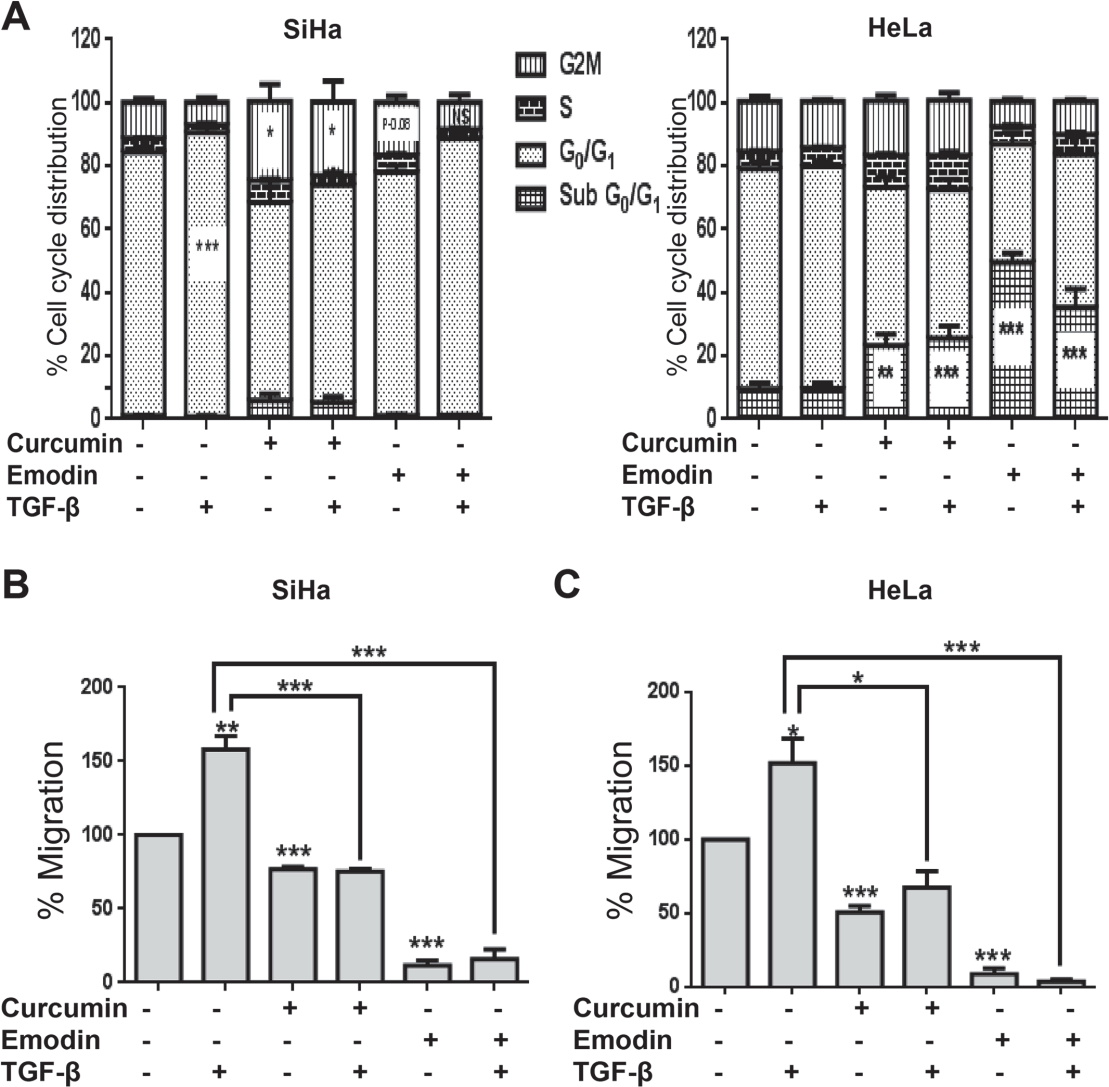
Effects of curcumin, emodin and TGF-β on cell cycle distribution and migration. SiHa and HeLa (A) cells were treated with curcumin or emodin in the presence or absence of TGF-β for 48h and subjected to cell cycle analysis using flow cytometry. Percentage distribution of different phases of cell cycle was plotted on the Y-axis against the indicated treatment conditions on the X-axis. SiHa (B) and HeLa (C) cells were seeded in a confluent monolayer. 16h after seeding, a scratch was made by a micro tip, and appropriate treatment was given in serum free medium. Scratched surface was imaged at 0h and 48h after treatment. The extent of migration in each sample was measured as the area covered by cells in 48h using TScratch software and represented as percentage change with respect to DMSO control (B) (n = 3, mean ± S.E.M.).

**Fig 4:**
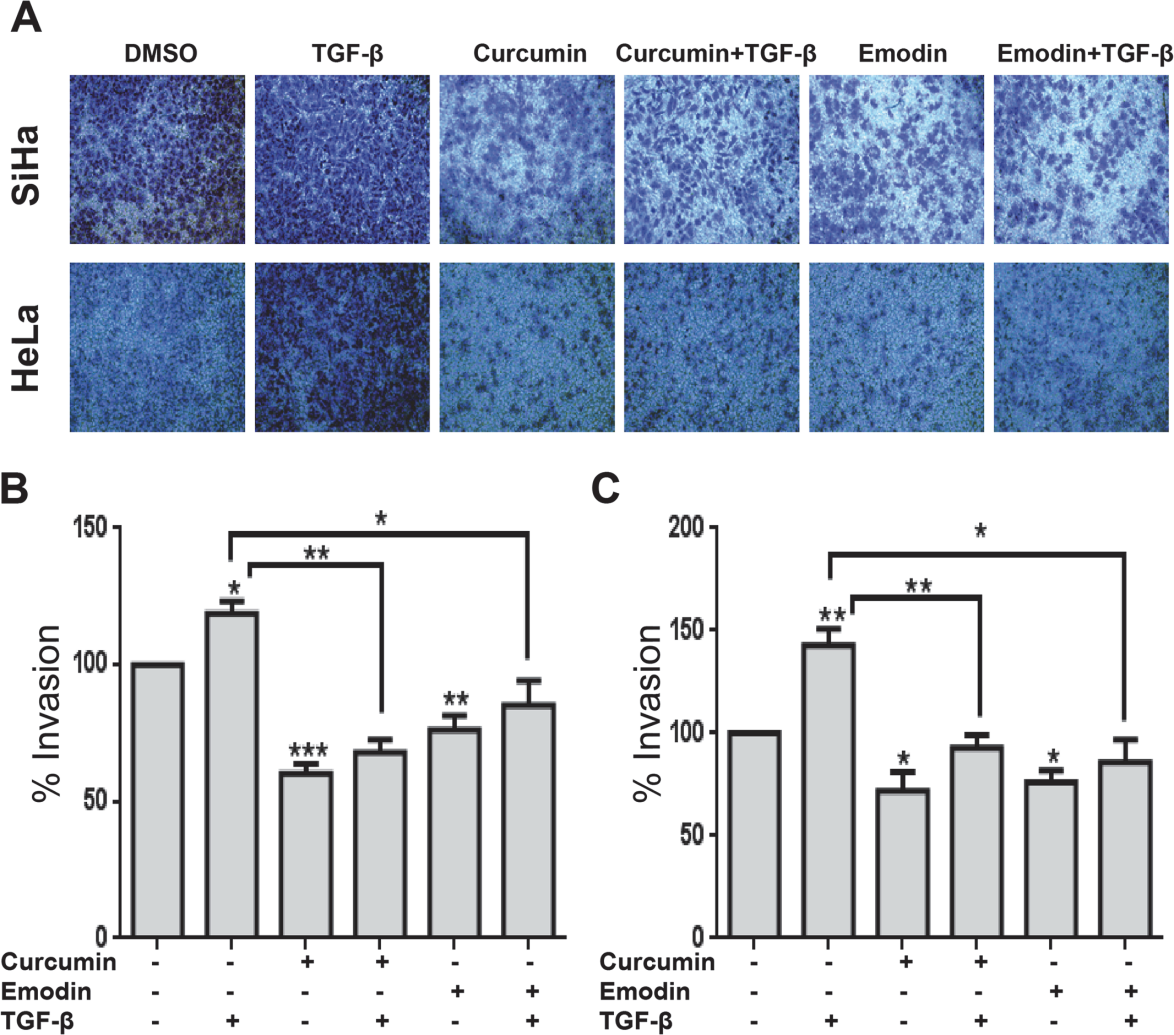
Effects of curcumin, emodin and TGF-β on cell invasion. SiHa (25,000) and HeLa (50,000) cells seeded onto Boyden chamber coated with matrigel, were treated with curcumin, emodin and/or TGF-β for 48h. Post treatment, cells were stained as described in Materials and Methods, and imaged using microscope (A). Quantification of invaded cells (B and C) was done by reading the absorbance of crystal violet at 595nm. Percentage invasion was calculated as fold change with respect to untreated control (n = 3, mean ± S.E.M.)

TGF-β is known to affect proliferation and migration during carcinogenesis [40]. We found that TGF-β induced G_0_/G_1_ arrest in SiHa, but not in HeLa cells (Fig. 3A) and the effect of curcumin and emodin on cell cycle profile under these conditions was further analyzed. Curcumin induced G_2_/M arrest in SiHa cells significantly, while emodin’s effect on G_2_/M phase was not statistically significant, though the arrest was visible (P-0.08). The presence of TGF-β did not significantly affect curcumin’s effect; while emodin-mediated partial G_2_/M arrest was found to be negated by TGF-β. However, both curcumin and emodin increased the sub G_0_/G_1_ population (indicative of apoptosis), in the presence as well as absence of TGF-β significantly in HeLa cells (Fig. 3A). Further, as observed by wound healing assay, TGF-β induced migration in both SiHa and HeLa cells and curcumin and emodin were found to inhibit the migration even in the presence of TGF-β (Fig. 3B and C). Furthermore, invasion assay using matrigel coated transwell inserts indicated that TGF-β induced invasion in both SiHa and HeLa cells, and curcumin and emodin inhibited both the basal as well of TGF-β induced invasion in these cells (Fig. 4). These results suggest that curcumin and emodin induce G_2_/M arrest in SiHa and apoptosis in HeLa cells, respectively. They significantly inhibit migration and invasion of SiHa and HeLa cells and are also able to counterbalance TGF-β induced migration and invasion in these cells.

### Curcumin and emodin inhibit TGF-β signaling pathway in SiHa and HeLa cells

**Fig 5:**
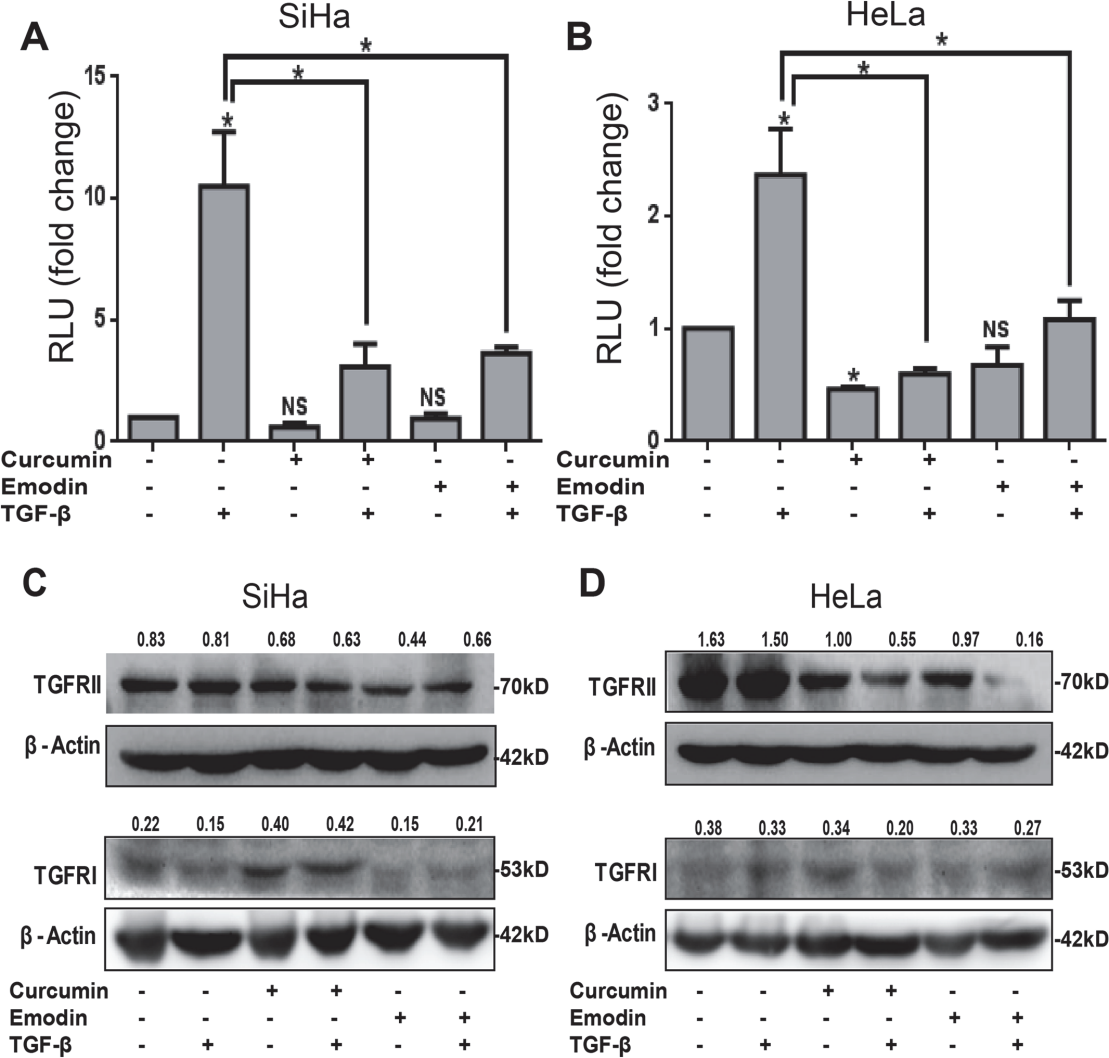
Effect of curcumin and emodin on Smad mediated gene transcription and expression of TGF-β receptors. SiHa (A) and HeLa (B) cells transfected with SBE firefly luciferase and TK-renilla luciferase constructs and treated with curcumin and emodin in presence or absence of TGF-β, were assayed for luciferase activity. Transfection efficiency was normalized by calculating the relative ratio of firefly to renilla luciferase activities and the results were plotted as fold change with respect to the untreated control. Representative experiment is shown, another independent experiment yielded similar results. Total cell lysates of SiHa (C) and HeLa (D) cells treated with curcumin or emodin in presence or absence of TGF-β for 48h and were subjected to immunoblotting with TGF-β Receptor I (TGFRI), TGF-β Receptor II (TGFRII) or β-Actin (loading control) antibodies. A representative blot is shown and the values shown represent densitometric analysis of the protein band with respect to β-Actin and similar results were confirmed in another independent experiment.

**Fig 6:**
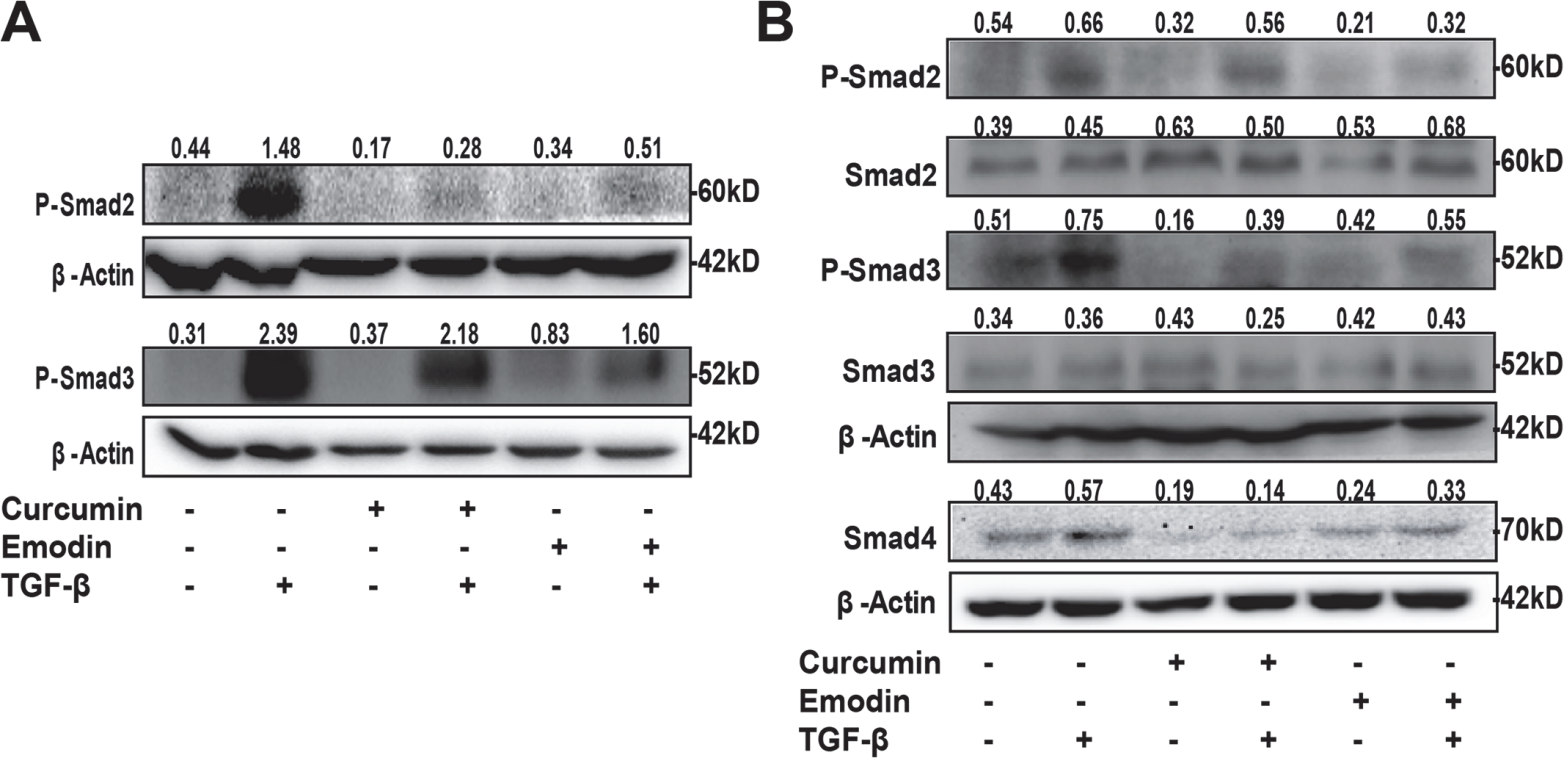
Effects of curcumin, emodin and TGF-β on the expression of Smad proteins. Total cell lysates of HeLa cells pretreated with curcumin and emodin for 48h, and treated with TGF-β for 30min was subjected to immunoblotting with P-Smad2 and P-Smad3 proteins (A). Total cell lysate of HeLa cells treated with curcumin or emodin, in presence or absence of TGF-β for 48h was subjected to immunoblotting with P-Smad2, P-Smad3, Smad2, Smad3, Smad4 and β-Actin (loading control) antibodies (B). A representative blot is shown and the values shown represent densitometric analysis of the protein band with respect to β-Actin and similar results were confirmed in another independent experiment.

To investigate whether these compounds would inhibit TGF-β signaling, TGF-β induced SBE luciferase activity in SiHa and HeLa cells was analyzed and found to be inhibited by both curcumin and emodin (Fig. 5A and B). To understand if TGF-β signaling is inhibited by affecting the expression of its receptors, we studied the effect of these compounds on the expression of TGF-β receptor proteins. As observed by western blotting, both in SiHa and HeLa cells, curcumin and emodin significantly down regulated the expression of TGF-β receptor II, while TGF-β receptor I was down regulated only in HeLa cells, and to a lesser extent (Fig. 5C and D). Although both SiHa and HeLa cells behaved similarly, the effects were more profound in HeLa cells and so they were chosen for further studies. In this regard, the expression of Smad proteins upon treatment with these compounds in the presence and absence of TGF-β was analyzed by western blotting. TGF-β is known to significantly induce the phosphorylation of Smad2 and Smad3 from 15min to one hour of treatment [41, 42], and thus the phosphorylation of Smad2 and Smad3 proteins was analyzed in HeLa cells pretreated with curcumin and emodin for 48h, and then treated with TGF-β for 30 min. TGF-β induced the phosphorylation of Smad2 and Smad3 significantly (Fig. 6A). Curcumin and emodin significantly inhibited the induction of phosphorylation of Smad2 and Smad3 in HeLa cells (Fig. 6A). Further, since the changes in expression of downstream targets of TGF-β and effectors related to EMT are generally observed by 24 to 72h [42], it was of interest to study the effect of co treatment of curcumin or emodin with TGF-β after 48h of TGF-β treatment on key players, downstream targets and effectors of TGF-β signaling pathway. At 48h of incubation also, TGF-β was found to induce the phosphorylation of Smad2/Smad3, and the expression of Smad4 protein and their basal as well as TGF-β-induced expression was down regulated by curcumin and emodin, while total Smad2 and Smad3 expression remained unaffected (Fig. 6B). Thus it was ascertained that curcumin and emodin down regulate TGF-β signaling pathway by down regulating TGF-β receptor II, P-Smad2, P-Smad3 and Smad4.

### Curcumin and emodin affect the downstream players and effectors of TGF-β Signaling pathway

**Fig 7:**
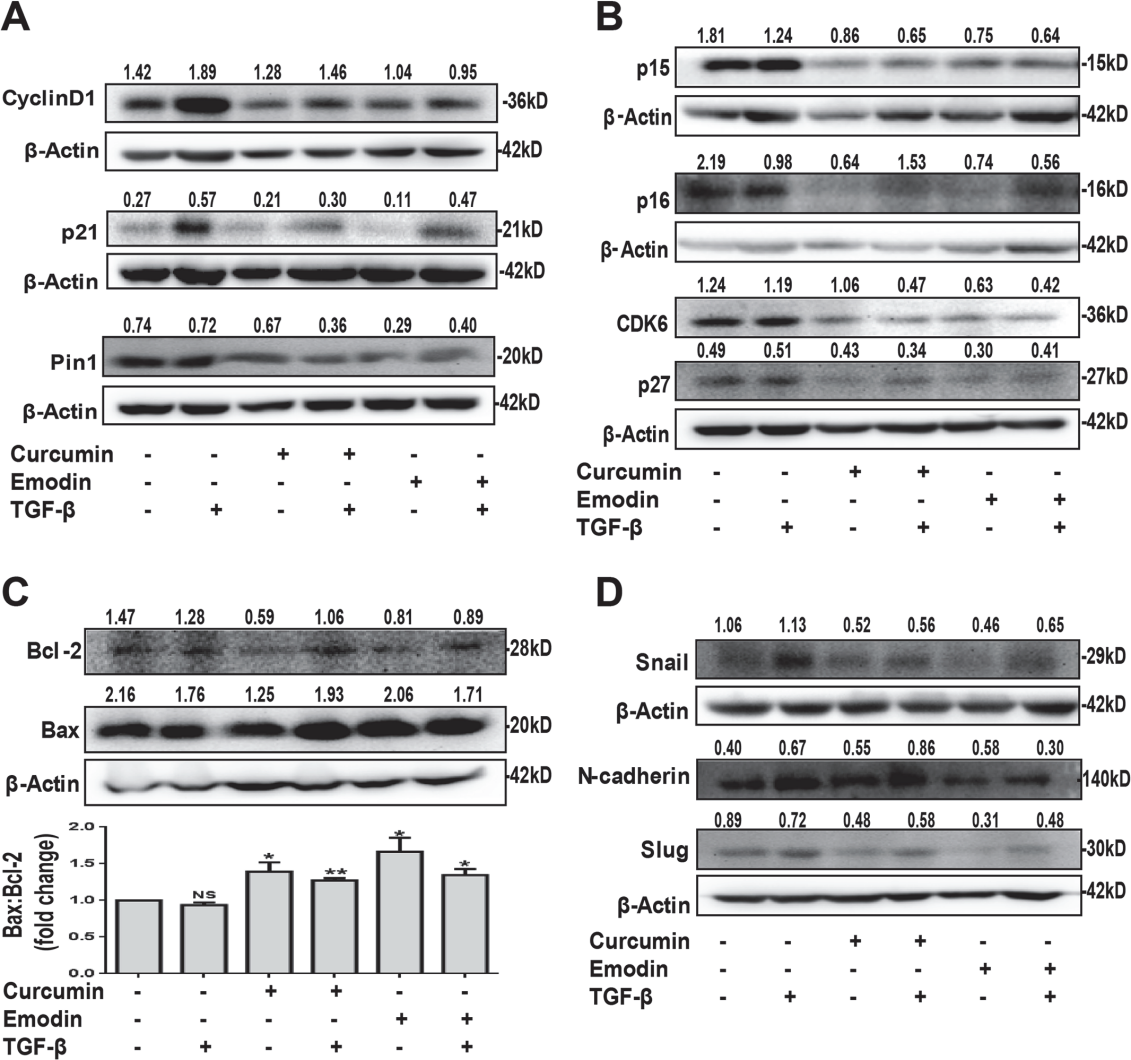
Effects of curcumin and emodin on EMT markers, downstream effectors of TGF-β signaling and Bax-Bcl-2. Total cell lysate of HeLa cells treated with curcumin or emodin, in presence or absence of TGF-β for 48h was subjected to immunoblotting with CyclinD1, p21, Pin1, β-Actin (A); p15, p16, CDK6, p27, β-Actin (B); Bcl-2, Bax, β-Actin antibodies, lower panel Bax: Bcl-2 densitometric analysis of the western blot (C) and Snail, N-cadherin, Slug, β-Actin (D). A representative blot is shown and the values shown represent densitometric analysis of the protein band with respect to β-Actin and similar results were confirmed in another independent experiment.

Smad-transcription factor complexes are known to regulate CDK6 inhibitor p21 [5]. We observed that the expression of p21 and CyclinD1 was found to be induced by TGF-β, but was down regulated upon curcumin and emodin treatment (Fig. 7A). TGF-β-induced migration is promoted by Pin1 [10] and as expected, curcumin and emodin down regulated Pin1 significantly (Fig. 7A). We found that curcumin and emodin significantly down regulated the expression of p15, p16, CDK6, p27 although curcumin could not inhibit p16 in the presence of TGF-β (Fig. 7B). Bax to Bcl-2 ratio suggests the susceptibility of cells to cell death [43] and it is up regulated upon curcumin and emodin treatment (Fig. 7C). These results suggest that curcumin and emodin block the cell cycle progression by inhibiting the expression of p15, p16, p27, CDK6, CyclinD1, p21, Pin1 and up regulating the ratio of Bax to Bcl-2 proteins.

Curcumin and emodin significantly down regulated the protein expression of Slug and Snail (key markers of metastasis and downstream effectors of TGF-β signaling pathway) (Fig. 7D). N-cadherin (mesenchymal marker) was down regulated significantly by emodin, but remained unaffected by curcumin (Fig. 7D). These results indicate that emodin could inhibit EMT by blocking the levels of Snail, Slug and N-cadherin, while curcumin mediated its effect mainly by inhibiting Snail and Slug.

### Curcumin and emodin inhibit Wnt/β-catenin signaling in the presence of TGF-β

**Fig 8:**
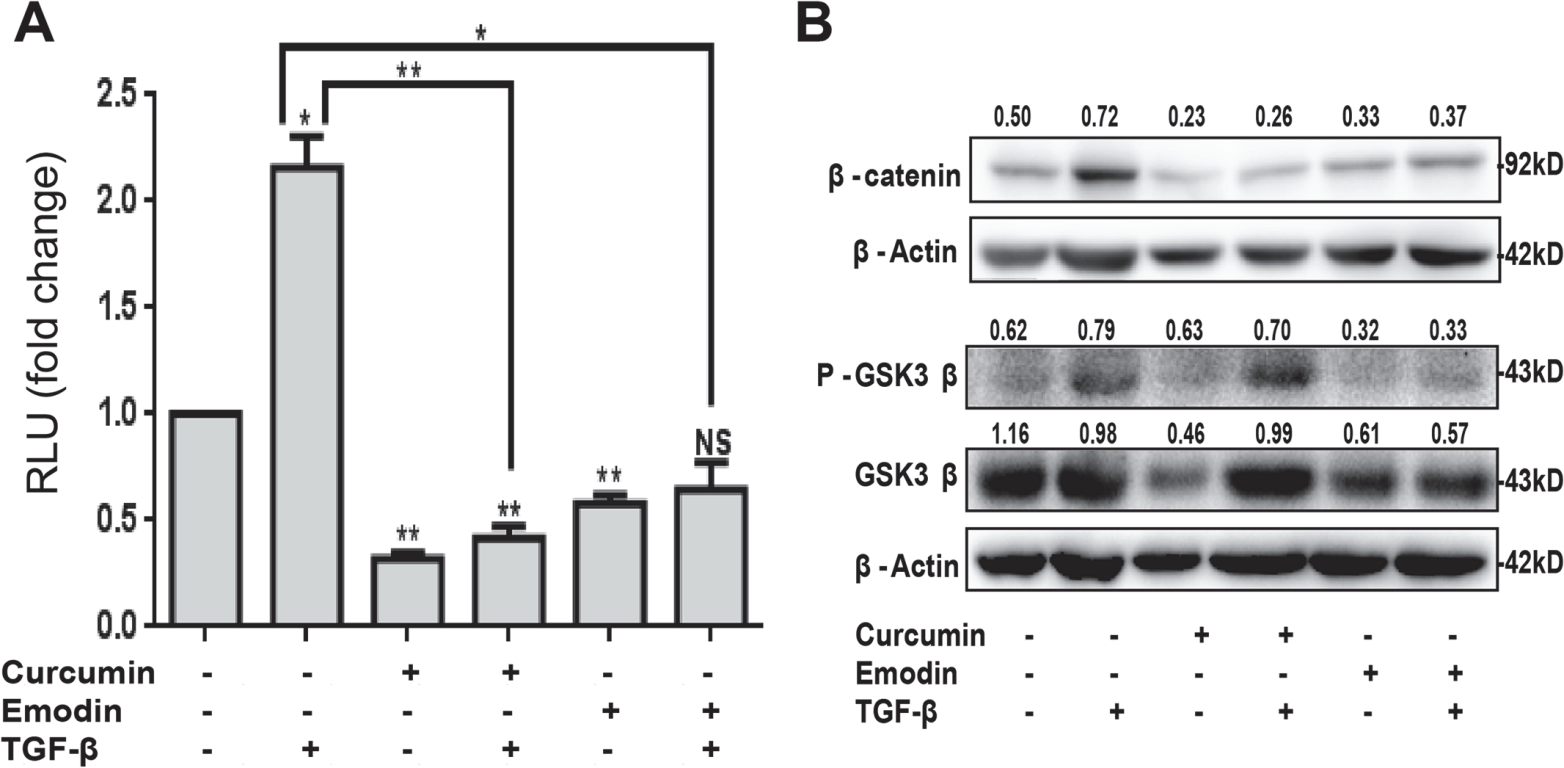
Effect of curcumin and emodin on Wnt/β-catenin signaling pathway in the presence or absence of TGF-β. HeLa cells were transfected with TOPFlash firefly luciferase and TK-Renilla luciferase constructs and treated with curcumin or emodin, in presence or absence of TGF-β and assayed for luciferase activity. Transfection efficiency was normalized by calculating the relative ratio of firefly to renilla luciferase activities and the results were plotted as fold change with respect to the untreated control (n = 3, mean ± SE). Total cell lysates of HeLa treated with curcumin or emodin in presence or absence of TGF-β for 48h were subjected to immunoblotting with β-catenin, P-GSK3β (ser9), GSK3β or β-Actin (loading control) antibodies (B). A representative blot is shown and the values shown represent densitometric analysis of the protein band with respect to β-Actin and similar results were confirmed in another independent experiment.

Wnt and TGF-β signaling pathways are known to have a cross talk, and also share common downstream targets [44]. Hence, it was of interest to investigate the effect of TGF-β on Wnt/β-catenin signaling in these cells, and study the effect of curcumin and emodin on Wnt/β-catenin signaling pathway. To this end, β-catenin-mediated transcriptional activation was analyzed by a read out TOPFlash luciferase reporter assay. TGF-β was found to induce TOPFlash luciferase activity suggesting activation of the Wnt/β-catenin signaling pathway, while curcumin and emodin down regulated it (Fig. 8A). Further, β-catenin (key player of the pathway) was found to be up regulated by TGF-β, and significantly down regulated upon curcumin and emodin treatment (Fig. 8B). P-GSK-3β (ser9) is inactive form of GSK-3β that leads to accumulation of β-catenin in the cell, was induced with TGF-β, and down regulated upon emodin treatment, also total GSK-3β protein levels were inhibited upon curcumin and emodin treatment, though the inhibition by curcumin was lost in the presence of TGF-β (Fig. 8B). These results suggest that TGF-β activates Wnt/β-catenin signaling pathway in HeLa cells, and curcumin and emodin down regulate the pathway by inhibiting β-catenin.

### Curcumin and emodin synergistically induce cytotoxicity in SiHa and HeLa cells

**Fig 9:**
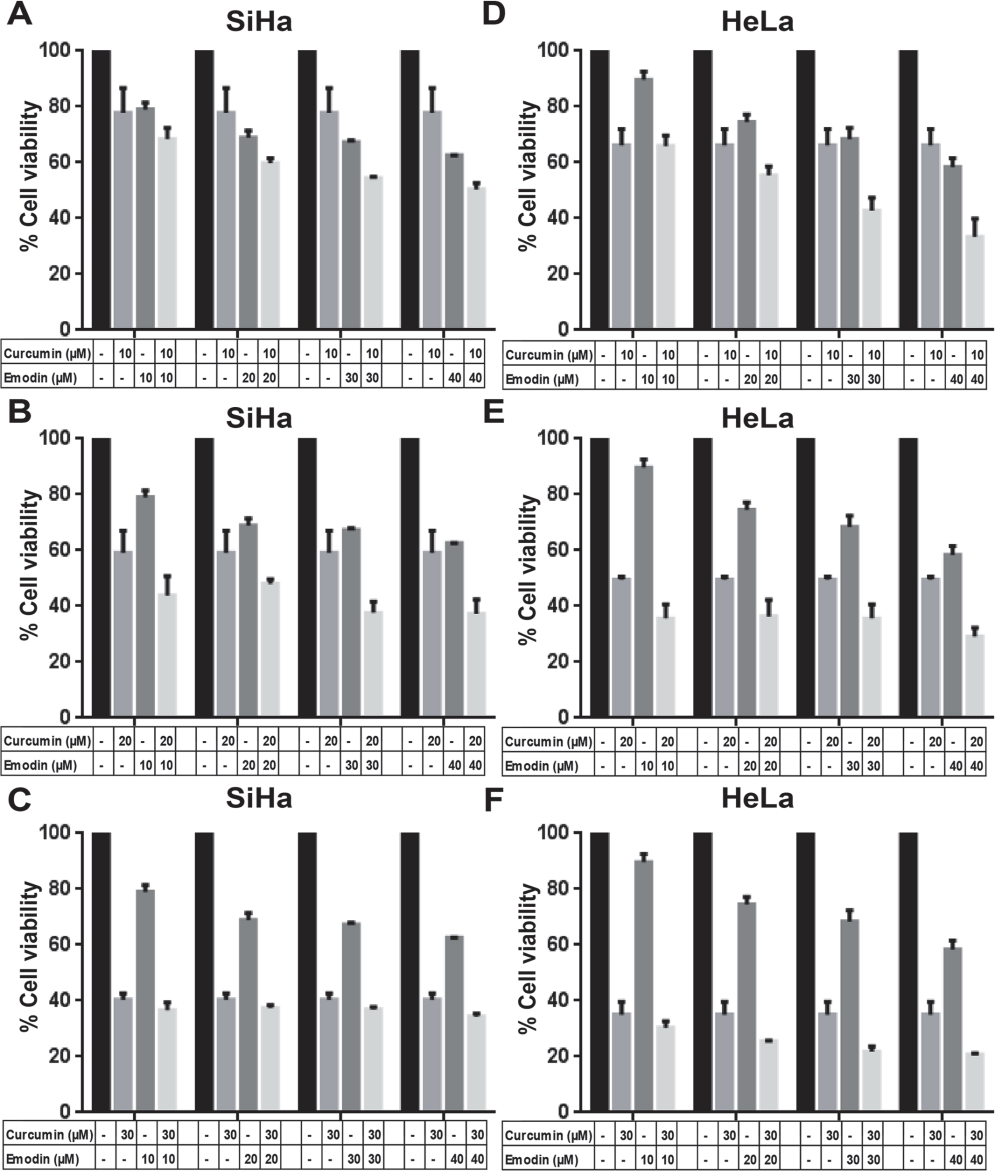
Changes in cell viability induced by different concentrations of curcumin and emodin in combination. SiHa (A,B,C) and HeLa (D,E,F) cells were treated with curcumin and emodin alone and in combination for 48h and assayed for cell viability by resazurin reduction method. Percentage cell viability was calculated by normalizing the absorbance of treated samples against the DMSO control (n = 3, mean ± S.E.M.).

**Fig 10:**
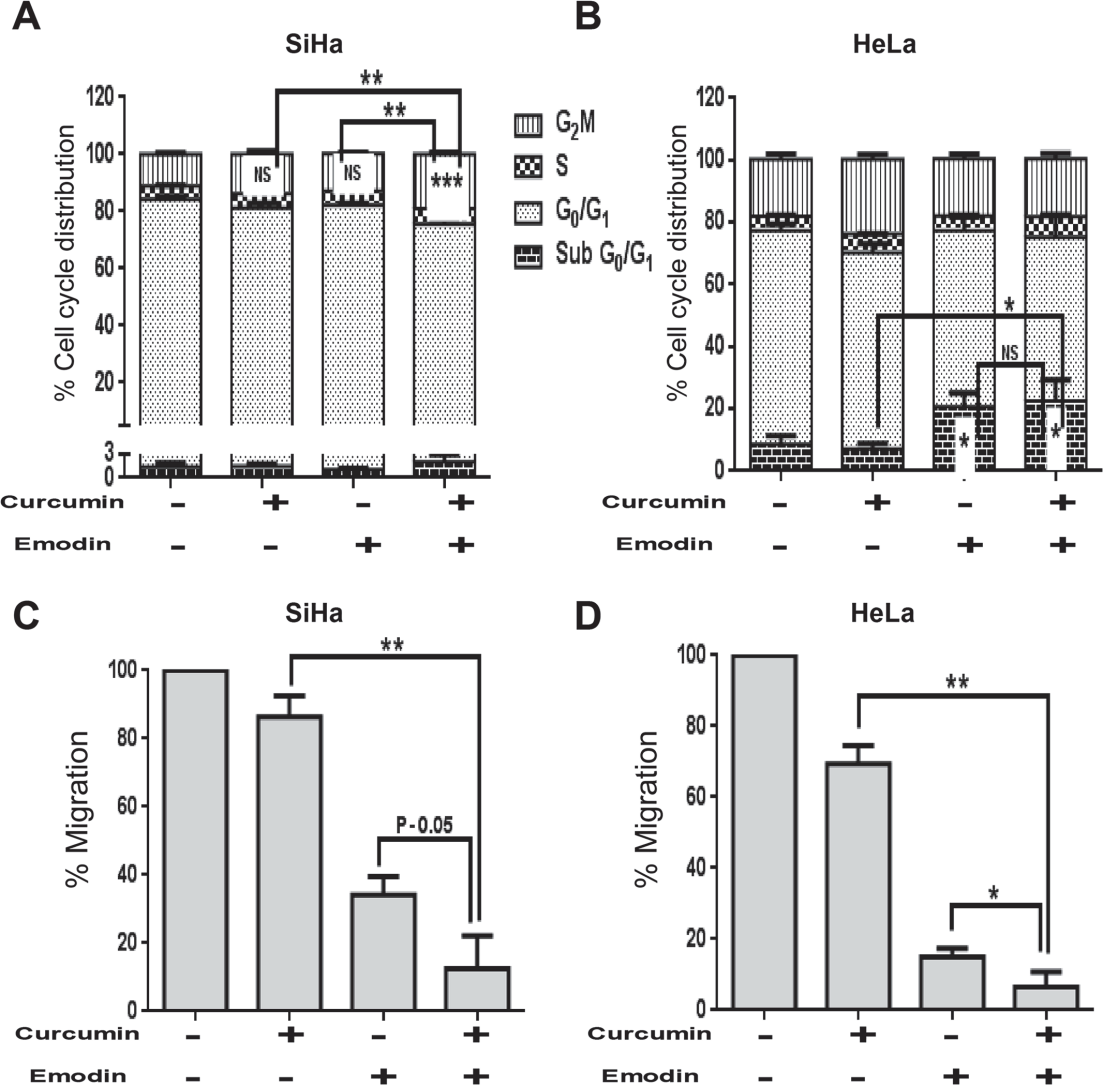
Combined effect of curcumin and emodin on changes in cell cycle distribution and migration. SiHa (A) and HeLa cells treated (B) with 10μM curcumin and 20μM emodin alone or in combination for 48h were subjected to cell cycle analysis using flow cytometry. Percentage distribution of different phases of cell cycle was plotted on the Y-axis against the indicated treatment conditions on the X-axis. SiHa (C) and HeLa (D) cells were seeded in a confluent monolayer. 16h after seeding, a scratch was made by a micro tip and cells treated with 10μM curcumin and 20μM emodin alone or in combination in serum free DMEM. Scratched surface was imaged at 0h and 48h after treatment. The extent of migration in each sample was measured as the area covered by cells in 48h using TScratch software and represented as fold change with respect to DMSO control (B) (n = 3, mean ± S.E.M.).

Since curcumin and emodin acted on similar targets, it was of interest to see if they could act synergistically. Different concentrations of emodin (10, 20, 30 and 40 μM) in combination with 10 (Fig. 9A and D), 20 (Fig. 9B and E) or 30μM (Fig. 9C and F) of curcumin were used to treat SiHa (Fig. 9A, B and C) and HeLa cells (Fig. 9D, E and F). Percentage cell viability was found to be lower in cells treated with the different combinations of curcumin and emodin used, than with either curcumin or emodin alone. Combination index (CI) was calculated using Chou-Talalay method, and CI<1 indicates synergy [45]. Interestingly, the CI was higher than one when a combination of 10μM curcumin and 10μM emodin was used but was less than one in both SiHa and HeLa cells irrespective of the other concentration combinations used (Table 2). A combination of 10μM curcumin and 20μM emodin (lowest concentration combination with CI<1) was chosen for further experiments. In SiHa cells, G_2_/M arrest was observed to be significant with the combinatorial treatment as compared to the cells treated with curcumin or emodin alone (Fig. 10A). HeLa cells showed significant subG_0_/G_1_ population upon emodin treatment, but no synergy was observed upon combinatorial treatment (Fig. 10A). These results indicate that the combinatorial treatment of curcumin and emodin resulted in a synergistic decrease in SiHa and HeLa cell population. SiHa cells showed increased growth arrest with the combination treatment, but the cell cycle profile upon combinatorial treatment was similar to that of emodin alone in HeLa cells.

**Table 2:** Combination index values for combinatorial treatment of curcumin and emodin in human cervical cancer cells.

Concentration μM		SiHa		HeLa	
Curcumin	Emodin	% Cell viability ± S.E.M.	CI	% Cell viability ± S.E.M.	CI
-	-	**100**		**100**	
**10**	-	**77.78±8.86**		**66.20±5.80**	
**20**	-	**59.25±7.77**		**49.54±1.04**	
**30**	-	**40.47±2.23**		**35.01±4.65**	
-	**10**	**79.01±2.39**		**89.71±2.83**	
-	**20**	**68.84±2.61**		**74.53±2.72**	
-	**30**	**67.29±0.70**		**68.42±4.01**	
-	**40**	**62.69±0.03**		**58.39±3.12**	
**10**	**10**	**68.47±4.00**	**1.11**	**65.93±3.75**	**1.1**
**10**	**20**	**59.81±1.75**	**0.96**	**55.35±3.31**	**0.92**
**10**	**30**	**54.39±0.59**	**0.89**	**42.76±4.73**	**0.93**
**10**	**40**	**50.45±2.20**	**0.84**	**33.43±6.41**	**0.84**
**20**	**10**	**43.94±6.89**	**0.77**	**35.61±5.06**	**0.79**
**20**	**20**	**48.03±1.68**	**0.73**	**36.45±5.82**	**0.82**
**20**	**30**	**37.71±3.97**	**0.71**	**35.68±5.05**	**0.86**
**20**	**40**	**37.29±5.13**	**0.74**	**29.23±3.07**	**0.95**
**30**	**10**	**36.74±2.69**	**0.9**	**30.44±2.18**	**0.85**
**30**	**20**	**37.42±1.02**	**0.96**	**25.62±0.21**	**0.82**
**30**	**30**	**36.99±0.78**	**0.99**	**21.75±1.95**	**0.78**
**30**	**40**	**34.47±0.96**	**0.94**	**20.83±0.27**	**0.84**

SiHa and HeLa cells treated with different concentration combinations of curcumin and emodin were assayed for cell viability by resazurin reduction method at 48h and combination indices (CI) were calculated from the changes in cell viability (mean of three experiments) using CompuSyn software.

### Combined treatment of curcumin and emodin suppresses cell migration, expression of EMT markers and downstream effectors of TGF-β signaling

**Fig 11:**
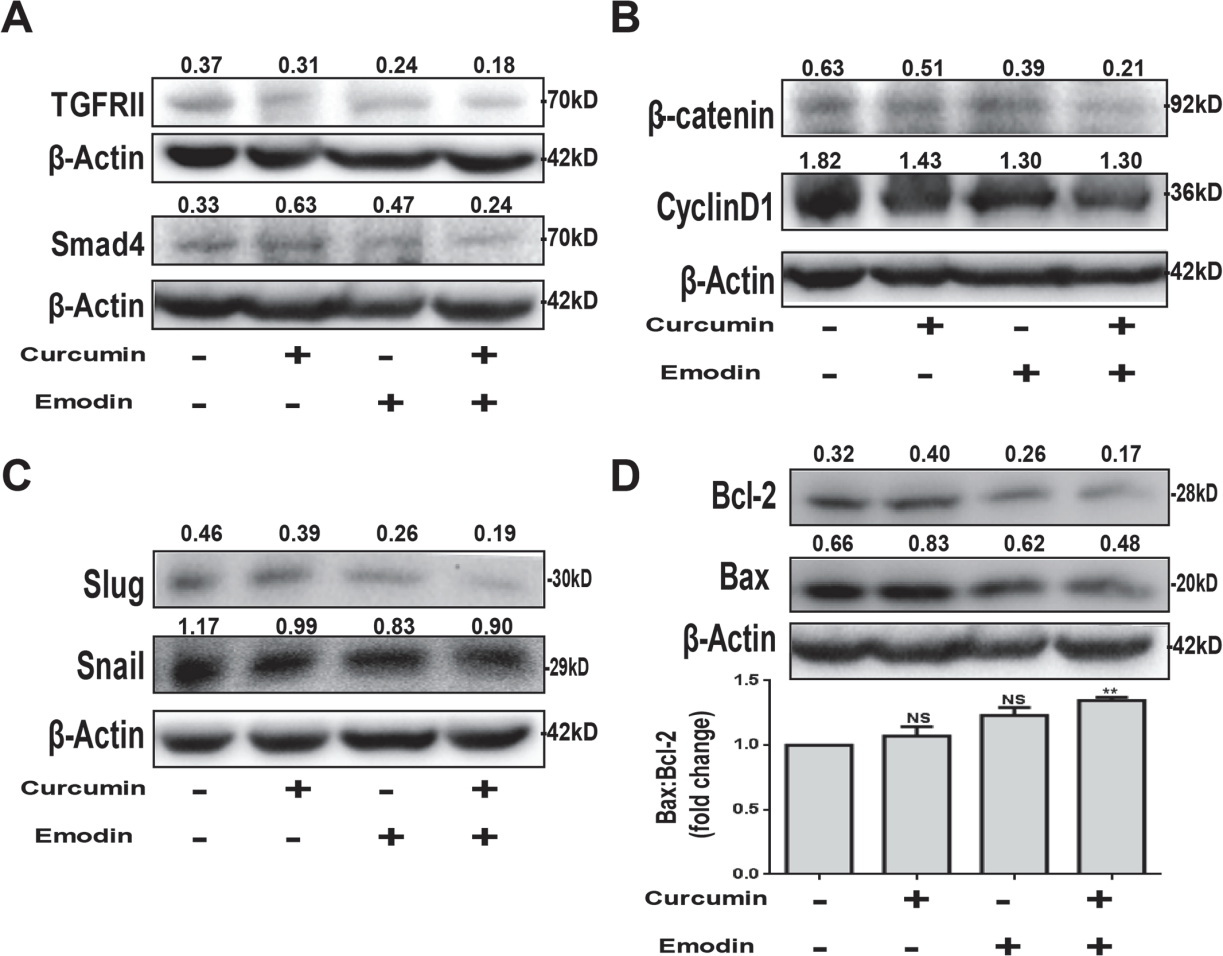
Combined effects of curcumin and emodin on EMT markers, effectors of TGF-β signaling and Bax-Bcl-2. Total cell lysate from HeLa cells treated with 10μM curcumin and 20μM emodin alone or in combination for 48h was subjected to immunoblotting with β-Actin (loading control), TGF-β receptor II (TGFRII), Smad4 (A), Slug, Snail (B), β-catenin, CyclinD1 (C) and Bax, Bcl-2 antibodies, lower panel Bax: Bcl-2 densitometric analysis of the western blot (D). A representative blot is shown and the values shown represent densitometric analysis of the protein band with respect to β-Actin and similar results were confirmed in another independent experiment.

Inhibition of migration in cells with the combination treatment was more pronounced than cells treated with curcumin or emodin alone (Fig. 10C and D). To further investigate the mechanistic basis of combination treatment, protein expression of EMT players and downstream effectors of TGF-β signaling were analyzed. Combination treatment was found to decrease the expression of TGF-β receptor II and Smad4 more significantly than either of them alone (Fig. 11A). Further β-catenin and Slug were down regulated more potently upon combination treatment, while the down-regulation of Snail and CyclinD1 was similar to that of emodin alone (Fig. 11B and C). Also the Bax to Bcl-2 ratio was more significantly up regulated upon the combinatorial treatment (Fig. 11D). These results suggest that the combined treatment of curcumin and emodin significantly down regulates migration of SiHa and HeLa cells, by the inhibition of TGF-β signaling.

### Combined treatment of curcumin and emodin down regulates cell growth, mitochondrial membrane potential and key players of TGF-β signaling pathway in presence or absence of TGF-β

**Fig 12:**
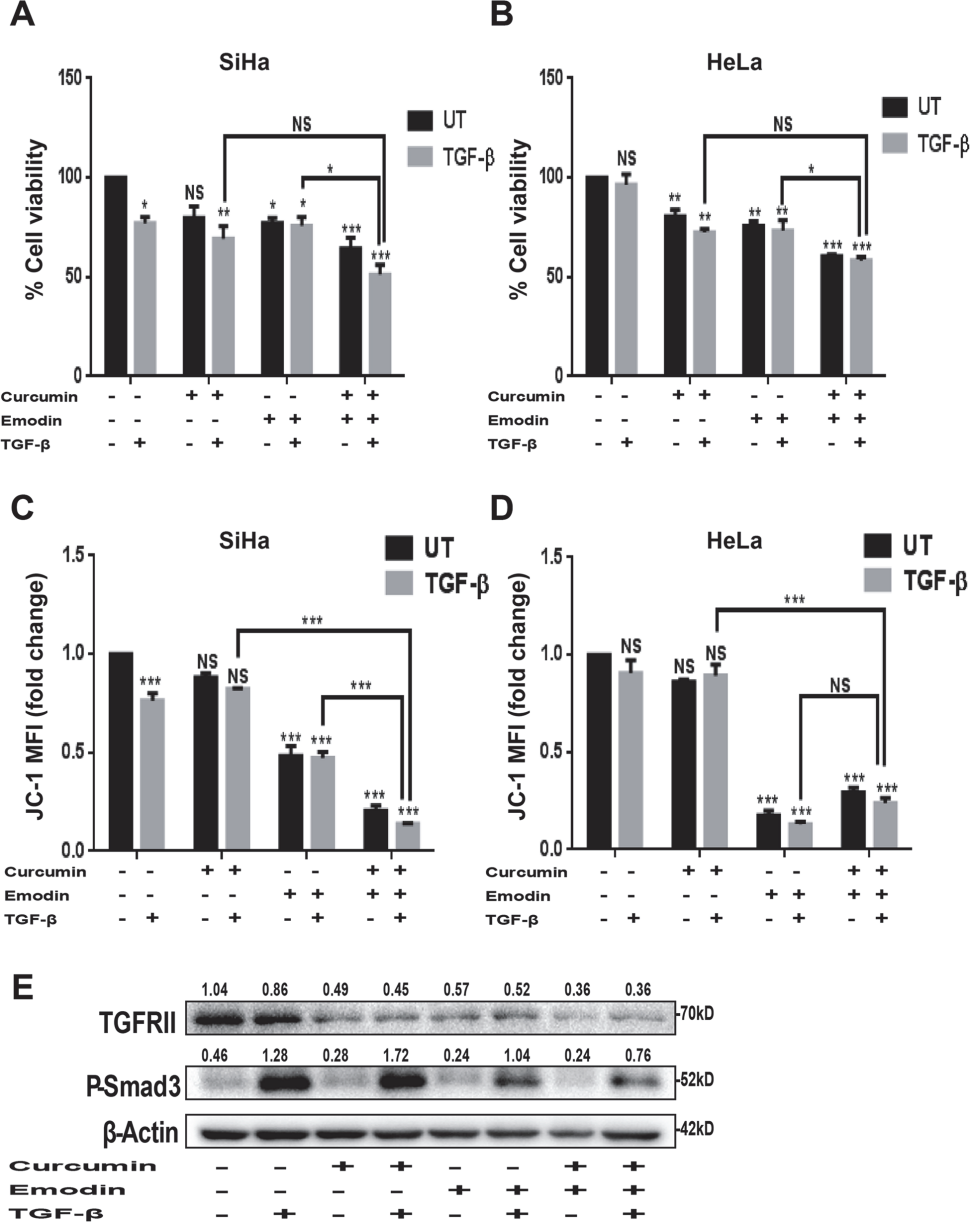
Combined effects of TGF-β, curcumin and emodin on viability, mitochondrial membrane potential and TGF-β signaling. SiHa (A and C) and HeLa cells treated (B and D) with 10μM curcumin and 20μM emodin alone or in combination in the presence or absence of TGF-β for 48h were assayed for cell viability (A and B) and mitochondrial membrane potential (C and D) (n = 3, mean ± S.E.M.). Total cell lysate from HeLa cells treated with 10μM curcumin and 20μM emodin alone or in combination in the presence or absence of TGF-β, for 48h was subjected to immunoblotting with TGF-β receptor II (TGFRII), P-Smad3 and β-Actin (loading control) antibodies (C). A representative blot is shown and the values shown represent densitometric analysis of the protein band with respect to β-Actin and similar results were confirmed in another independent experiment.

Since the combination of curcumin and emodin down regulated the expression of downstream effectors of TGF-β signaling pathway, it was of interest to study their combinatorial effect on cytotoxicity as well as key players of TGF-β signaling pathway in the presence of TGF-β. The combinatorial treatment of curcumin and emodin resulted in decrease of cell viability and mitochondrial membrane potential in SiHa and HeLa cells; however the inhibition of cell viability upon combinatorial treatment in presence of TGF-β was not significantly higher than cells treated with curcumin plus TGF-β in both SiHa and HeLa cells (Fig. 12A and B). Nevertheless, inhibition of mitochondrial membrane potential was enhanced significantly upon the combinatorial treatment even in the presence of TGF-β in SiHa cells, while the effect was not further potentiated in HeLa cells (Fig. 12C and D). Further, the inhibition of the expression of TGFβ-Receptor II and phosphorylated Smad3 was found to be higher upon combinatorial treatment compared to cells treated with curcumin or emodin individually both in presence as well as absence of TGF-β (Fig. 12E). These results indicate that combinatorial treatment of curcumin and emodin enhanced the cytotoxicity in presence as well as absence of TGF-β, although the synergistic or additive effects were not significant in the presence of TGF-β. Combinatorial treatment of curcumin and emodin also enhanced their inhibitory effects on TGF-β signaling through inhibition of TGF-β Receptor II expression and phosphorylation of Smad3.

## Discussion

High-risk HPV infection together with a plethora of cellular, immunological, genetic, epigenetic and environmental factors affect the occurrence and progression of cervical carcinoma [2]. TGF-β down regulates the expression of HPV16 E6 and E7 oncogenes and cells, which were transformed by such oncogenes or cultured over several passages show partial resistance to the inhibitory effects of TGF-β [46]. HPV 16 E7 protein is shown to block the ability of TGF-β to inhibit cell proliferation [47] suggesting an association of HPV-associated malignant transformation to the loss of responsiveness to the TGF-β-mediated growth inhibitory signal. Cervical cancer cells secrete high levels of TGF-β that inhibit tumor infiltration by apoptosing CD4 lymphocytes evading host immune system [17]. Moreover, high expression of TGF-β1 predicts poor prognosis in patients with stage Ib-II1 squamous cell carcinoma [48]. These studies indicate that additional molecular changes, post HPV infection, participate in the loss of TGF-β responsiveness promoting malignant transformation. Phytochemicals, known to antagonize factors deregulated in malignant cells, are considered as good potential candidates for chemoprevention and therapy [19]. Significant inhibition of cell viability and mitochondrial membrane potential combined with the induction of cell cycle arrest by curcumin and emodin in cervical cancer cells are consistent with earlier reports [24, 26–28, 49]. We observed that TGF-β induces migration and invasion in both SiHa and HeLa cells which is in line with its widely established role as an inducer of EMT contributing to invasive, migratory and stem cell properties of tumor cells allowing them to metastasize [9, 40]. SiHa and HeLa cells responded differentially to TGF-β, wherein SiHa cells showed mild growth arrest, while HeLa cells remained unaffected [39]. Although SiHa cells were shown to be resistant to TGF-β due to mutation in Smad4 [50], we did not detect any mutations in Smad4 in these cells [51] for which the actual reasons are not clear but could be due to experimental and/or genetic variations. Nevertheless, TGF-β was shown to induce epithelial to mesenchymal transition in SiHa cells in line with our results [52]. This is the first report showing down regulation of TGF-β signaling pathway by curcumin and emodin in cervical cancer cells and their effect was independent of the presence of TGF-β suggesting their suitability to combat carcinogenesis during early as well as advanced stages of cancer wherein expression and secretion of TGF-β are differentially regulated [53]. Inhibition of TGF-β signaling by down-regulation of TGF-β ReceptorII and alterations in the expression of Snail, Twist, Vimentin, E-cadherin, P-Smad2, β-catenin and GSK3-β by curcumin in triple negative breast cancer cells suppressed EMT [54]. Curcumin also down-regulated TGF-β signaling cascade in breast and pancreatic cancer cells by inducing apoptosis and reversing EMT [30, 55]. Surprisingly, curcumin reduced subcutaneous tumor growth in lung cancer cells independent of their TGF-β status, while abrogating TGF-β signaling by inhibiting TGF-β-induced Smad2 and Smad3 phosphorylation in TGF-responsive H358 and A549 cells, but not in ACC-LC-176 cells wherein TGF-β signaling was non-functional [56]. Effects of TGF-β may be mediated through several regulators of cell cycle and may thus depend on the context or cell-type. HPV16 E7 oncogene can bind and inactivate p27 without degrading it in human cervical cancer cells [57]. Activation of the Ras/MAPK pathway enhances the mitogenic action of TGF-β by suppressing its growth-inhibitory function and inhibition of p15^INK4B^ or p21^WAF1^ [58]. Notch signaling activation blocks the TGF-β-dependent upregulation of the cyclin-dependent kinase (CDK) inhibitors p15, p16, p21 and p27, whereas its inhibition induces them [59]. Our observation of inhibition of CyclinD1 and CDK6 by curcumin is in line with an earlier report showing curcumin-mediated increase in acetylation and up-regulation of p53 leading to cell cycle arrest at G_1_-S phase [60]. Inhibition of CyclinD1 and CDK4 by curcumin was observed in cervical cancer cells, although it was accompanied by up-regulation of p21, p27 contradicting our data showing down-regulation of p21, and p27 [60] suggesting the complexity of its action involving mediators other than p21 and p27. Down regulation of p21, CyclinD1, CDK6 and Pin1 by curcumin and emodin highlight their growth inhibitory potential. TGF-β-mediated CyclinD1 and p21 gene expression increases breast cancer migration and invasion *in vitro* [61]. p21 can also promote oncogenesis independently of its anti-apoptotic activity by promoting the assembly of complexes of CyclinD with CDK4 or CDK6 [62]. While Pin1 elevates TGF-β induced EMT, it is also shown to negatively regulate TGF-β signaling by down-regulating Smad2/3 protein levels, reducing TGF-β-mediated induction of N-cadherin [10, 63]. Given that the expression of p15 and p16 (tumor suppressors) was down-regulated upon curcumin and emodin treatment in cervical cancer cells, their growth inhibitory effects may have taken an alternate route through Bax induction together with the down regulation of Bcl-2, but whether curcumin and emodin influence other proteins involved in the tumor suppressive role of TGF-β needs to be deciphered. While there are no reports of emodin-mediated effect on TGF-β signaling in cancer cells, emodin is known to induce tissue regeneration and wound healing of fibroblasts by up-regulating Smad-mediated TGF-β signaling pathway [32, 64]. Peculiarly, emodin suppressed tumor necrosis factor -α induced fibroblast migration and fibronectin deposition *in vitro* without affecting TGF-β signaling [65]. Also emodin is widely known to block invasion, migration and metastasis in multiple systems [66–68].

TGF-β had an activating effect on Wnt/β-catenin signaling pathway and curcumin and emodin inhibited the induction, suggesting these compounds act by coordinated action of multiple pathways. Wnt/β-catenin and TGF-β signaling pathways are known to have a cross talk and thereby influence cervical tumorigenesis [69, 70]. TGF-β is known to stimulate CyclinD1 expression through activation of Wnt/β-catenin signaling, at least in part wherein SMAD3 and β-catenin co-localized to the nucleus after TGF-β treatment [11, 44]. However, further investigation is needed on the effect of curcumin and emodin on TGF-β mediated effects on Wnt, RAS, MAPK and TNF-α pathways. Consistent with our data, combined curcumin and emodin administration was shown to synergistically inhibit proliferation, survival and invasion of breast cancer cells where the antitumor effects were mediated through miR-34a by down regulating Bcl-2 and Bmi [71]. Our data on the synergistic action of curcumin and emodin indicating enhanced effects on cell viability and migration in both HeLa and SiHa cells suggest that the combinatorial effect counterbalances the weaker effects of individual drug, as the stronger effect (G_2_M arrest in HeLa cells) was not further potentiated. However, further studies with different combination treatments are warranted to confirm it. The major stumbling block towards usage of curcumin and emodin is their poor bioavailability more because of our limited understanding of their absorption and metabolism *in vivo* [72, 73]. However, a significant step towards taking curcumin to clinic, Vacurin amphipathic vaginal cream—a uniform colloidal solution of curcumin, was shown to selectively eliminate a variety of HPV (+) cervical cancer cells, suppress the transforming antigen E6 and concomitantly induce p53, suppressing expression of EGFR [74]. TGF-β paradox-switch of TGF-β signaling from tumor suppressor to oncogenic pathway, calls for identification of new drugs and their targets that block the tumor progressive effects of TGF-β without reacting to its growth inhibitory activity. Our findings suggest that curcumin and emodin inhibit growth and migration while down regulating TGF-β signaling pathway in cervical cancer cells. Thus the study contributes to better understanding of the mechanistic basis of action of curcumin and emodin, and proposes therapeutic strategies to inhibit cell proliferation and migration and to activate apoptosis to fight cervical cancer.
